# Exercise-induced dehydration alters pulmonary function but does not modify airway responsiveness to dry air in athletes with mild asthma

**DOI:** 10.1152/japplphysiol.01114.2016

**Published:** 2017-03-09

**Authors:** A. J. Simpson, L. M. Romer, P. Kippelen

**Affiliations:** Centre for Human Performance, Exercise, and Rehabilitation, Division of Sport, Health, and Exercise Sciences, College of Health and Life Sciences, Brunel UniversityLondon, Uxbridge, United Kingdom

**Keywords:** airway hyperresponsiveness, eucapnic voluntary hyperpnea, exercise-induced bronchoconstriction, exercise-induced asthma, whole body dehydration

## Abstract

Local airway water loss is the main physiological trigger for exercise-induced bronchoconstriction (EIB). Our aim was to investigate the effects of whole body water loss on airway responsiveness and pulmonary function in athletes with mild asthma and/or EIB. Ten recreational athletes with a medical diagnosis of mild asthma and/or EIB completed a randomized, crossover study. Pulmonary function tests, including spirometry, whole body plethysmography, and diffusing capacity of the lung for carbon monoxide (Dl_CO_), were conducted before and after three conditions: *1*) 2 h of exercise in the heat with no fluid intake (dehydration), *2*) 2 h of exercise with ad libitum fluid intake (control), and *3*) a time-matched rest period (rest). Airway responsiveness was assessed 2 h postexercise/rest via eucapnic voluntary hyperpnea (EVH) to dry air. Exercise in the heat with no fluid intake induced a state of mild dehydration, with a body mass loss of 2.3 ± 0.8% (SD). After EVH, airway narrowing was not different between conditions: median (interquartile range) maximum fall in forced expiratory volume in 1 s was 13 (7–15)%, 11 (9–24)%, and 12 (7–20)% in dehydration, control, and rest conditions, respectively. Dehydration caused a significant reduction in forced vital capacity (300 ± 190 ml, *P* = 0.001) and concomitant increases in residual volume (260 ± 180 ml, *P* = 0.001) and functional residual capacity (260 ± 250 ml, *P* = 0.011), with no change in Dl_CO_. Mild exercise-induced dehydration does not exaggerate airway responsiveness to dry air in athletes with mild asthma/EIB but may affect small airway function.

**NEW & NOTEWORTHY** This study is the first to investigate the effect of whole body dehydration on airway responsiveness. Our data suggest that the airway response to dry air hyperpnea in athletes with mild asthma and/or exercise-induced bronchoconstriction is not exacerbated in a state of mild dehydration. On the basis of alterations in lung volumes, however, exercise-induced dehydration appears to compromise small airway function.

## Abstract (precis)

This study is the first to investigate the effect of whole body dehydration on airway responsiveness. Our data suggest that the airway response to dry air hyperpnea in athletes with mild asthma and/or exercise-induced bronchoconstriction is not exacerbated in a state of mild dehydration. On the basis of recorded alterations in lung volumes, however, exercise-induced dehydration appears to compromise small airway function.

whole body dehydration commonly occurs in athletes engaging in endurance events (32), with loss of body mass frequently averaging 2–3% (31, 37). Whole body dehydration is thought to limit exercise performance due to strain on multiple organ systems, including the circulatory, (central) nervous, muscular, integumentary, and urinary systems (8, 33). Lung fluid balance and water transport at pulmonary surfaces play an important physiological role in the maintenance of airway hydration and in proper airway clearance (16). Relatively little is known about the impact of whole body dehydration on the respiratory system.

Only two studies have specifically investigated the effect of whole body dehydration on pulmonary function (13, 15). A reduction in forced expiratory volume in 1 s (FEV_1_) was noted in mildly dehydrated individuals following fluid deprivation (13). However, after diuretic (chlortalidone) drug administration, resulting in moderate dehydration, an increase in expiratory flow rates (including FEV_1_) was noted (15). Therefore, uncertainty remains as to the impact of whole body dehydration on the healthy human lung.

Equally uncertain is whether whole body dehydration constitutes a significant risk factor for bronchopulmonary disorders (16). A large body of evidence points to acute dehydration of the airway surface liquid as a key determinant of exercise-induced bronchoconstriction (EIB) (2). EIB is characterized by a transient narrowing of the airways (with associated reduction in expiratory airflow) in response to vigorous exercise. Individuals most at risk for EIB are endurance athletes and patients with asthma (11, 17). During exercise-induced hyperpnea, water and heat are lost from the airway surface in response to humidification of large volumes of inspired (unconditioned) air over a short period of time (10). The evaporative water loss is proposed to increase the osmolarity of the airway surface liquid, particularly at the level of the small airways (9). This would then stimulate the release of bronchoactive mediators and cause, in susceptible individuals, the airway smooth muscle to contract (2).

The primary provider of fluid to the airways is the bronchial circulation. Since exercise-induced dehydration causes hypovolemia and increases blood plasma osmolarity (8), alterations in the volume and composition of bronchial blood flow are to be expected in a state of dehydration. Whole body dehydration may therefore diminish airway surface hydration, resulting in an amplified bronchoconstrictive response to exercise in individuals with EIB.

The primary aim of this study was to establish the impact of whole body dehydration, induced by prolonged exercise in the heat, on airway responsiveness in athletes with a prior medical diagnosis of mild asthma and/or EIB. Our hypothesis was that the fall in FEV_1_ after dry air hyperpnea would be exacerbated in a state of mild dehydration. Since the effect of whole body dehydration on resting pulmonary function remains uncertain, we also assessed pulmonary function, via spirometry, whole body plethysmography, and diffusing capacity of the lung for carbon monoxide (Dl_CO_), before and after induced dehydration.

## METHODS

### Participants

Ten recreational athletes (4 women) completed the study. Mean age, height, and body mass were 21 ± 2 yr, 170 ± 12 cm, and 63 ± 10 kg, respectively. Participants were involved in summer sports and trained for 6 ± 4 h/wk in aerobic activities. All participants had a prior medical diagnosis of mild asthma and/or EIB and reported respiratory symptoms (chest tightness, wheeze, mucus hypersecretion, and cough) during and/or after exercise. Five participants had childhood asthma, and eight were using short-acting β_2_-agonist medication. Participants taking any asthma medication other than inhaled short-acting β_2_-agonists or antihistamines were excluded. Those on medication(s) were required to withhold inhaled short-acting β_2_-agonists for ≥8 h and antihistamines for 72 h before each visit (4). All participants had baseline FEV_1_ and forced vital capacity (FVC) above the lower limit of normal (30). Participants were nonsmokers, were free from respiratory infection for 4 wk before the study, and had no known chronic medical condition other than asthma or EIB. All participants provided written informed consent. The institutional research ethics committee approved the study (reference no. RE52-12).

### Protocol

**Fig. 1.**
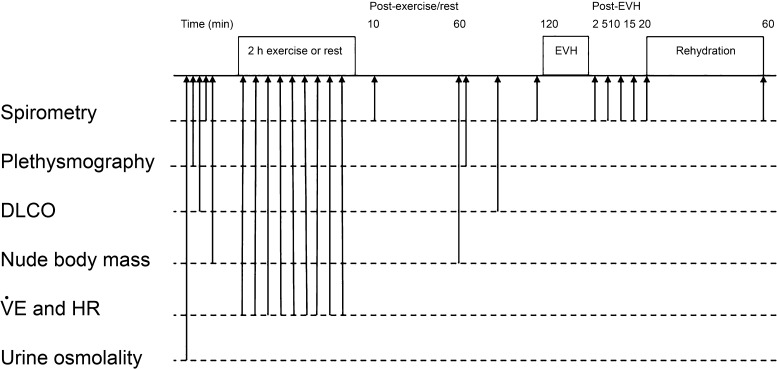
Schematic of protocol to assess changes in airway responsiveness and pulmonary function in a dehydration condition (2 h of exercise in the heat with fluid restriction), a control condition (2 h of exercise in ambient conditions with voluntary fluid consumption), and a time-matched rest condition (2 h of rest with voluntary fluid consumption). EVH, eucapnic voluntary hyperpnea; Dl_CO_, diffusing capacity of the lung for carbon monoxide; V̇_E_, ventilation; HR, heart rate.

The study used a randomized crossover design with three experimental visits. The order of the experimental visits was randomized using the random number generator function in Microsoft Excel (2011), and visits were separated by >48 h. Pulmonary function was assessed using spirometry, whole body plethysmography, and diffusing capacity before and up to 2 h after each of the following conditions: *1*) exercise in the heat with no fluid intake (dehydration), *2*) exercise with ad libitum fluid intake (control), and *3*) a time-matched rest period (rest). To avoid influence of airway refractoriness (21), exercise intensity was set low (see *Exercise*) and airway responsiveness was assessed 2 h after exercise. The “rest” condition was included to further ensure that a refractory response was not present at the time of the bronchial challenge with dry air. To determine whether any changes caused by dehydration could be quickly reversed, a rehydration phase was included in the dehydration condition. In that condition, participants were allowed to drink water ad libitum between 20 and 60 min after the eucapnic voluntary hyperpnea (EVH) challenge, after which final spirometry testing was performed. A schematic of the experimental protocol is presented in Fig. 1.

All visits commenced in the morning so as to standardize for diurnal variation in pulmonary function (34). Participants were asked to abstain from alcohol, caffeine, and exercise on the day of testing.

#### Hydration status.

Participants were asked to arrive at each experimental visit in a euhydrated state. Upon arrival, urine osmolality was measured using a portable refractive index osmometer (Osmocheck, Vitech Scientific, Horsham, UK). Adequate hydration was defined as <700 mosmol/kgH_2_O (32). Nude body mass was recorded before and 60 min after exercise or time-matched rest using a calibrated scale (model 798, Seca, Hamburg, Germany), with the change in body mass used as the index of dehydration.

#### Exercise.

In the control and dehydration conditions, participants completed 2 h of low-intensity exercise. The exercise involved four 20-min bouts of cycling, with each bout followed by 10 min of stepping. Cycling was performed at 25% of estimated peak power (14). Stepping was conducted on a 20-cm step at a rate of 45 steps/min. Midway through each bout of exercise, heart rate was measured using telemetry (Polar H7, Polar Electro, Warwick, UK) and minute ventilation was determined by offline gas analysis (Douglas bags and Harvard dry gas meter). To induce dehydration, exercise was performed in an environmental chamber (Procema, Twickenham, UK) set at 37°C and 50% relative humidity, and fluid intake was prohibited. In the control condition, environmental temperature was set at 20°C (ambient humidity), and subjects were allowed to consume fluid ad libitum. In the rest condition, participants remained seated in ambient conditions and were allowed to consume fluid ad libitum.

### Pulmonary Function

Pulmonary function was assessed using a commercially available system (Masterscreen, CareFusion, Hochberg, Germany). Spirometry was conducted at baseline and at 10 and 120 min after exercise (or rest). Forced expiratory maneuvers were performed in accordance with American Thoracic Society/European Respiratory Society (ATS/ERS) guidelines (25). Measurements were performed in triplicate, and the largest FEV_1_ and FVC from reproducible maneuvers (i.e., between-maneuver differences <150 ml for FEV_1_ and FVC) were kept for analysis. After the EVH challenge, expiratory maneuvers were performed in duplicate (1). The Global Lungs Initiatives GLI 2012 equations (30) were used for calculation of predicted values and lower limits of normal.

Whole body plethysmography was used to determine static lung volumes and capacities according to ATS/ERS guidelines (35). Measurements were performed at baseline and 60 min after exercise (or rest). The mean of three reproducible trials [i.e., the 3 functional residual capacity (FRC) values agreeing within 5%] was used for analysis. Residual volume (RV) was derived from the mean FRC minus mean expiratory reserve volume (ERV), and total lung capacity (TLC) was calculated as the sum of maximum vital capacity (VC) and RV.

Dl_CO_ was assessed using the single-breath technique according to ATS/ERS guidelines (23). The measurements were performed at baseline and 90 min after exercise (or rest). The maneuver was repeated at least twice to ensure repeatability (i.e., <10% variation in Dl_CO_). The mean Dl_CO_, transfer coefficient (*K*_CO_), and alveolar volume (Va) were calculated from two reproducible maneuvers and used for analysis. Diffusing capacity data for one participant were lost due to technical error.

### Airway Responsiveness

Airway responsiveness to dry air was assessed via EVH (1). Briefly, participants were asked to breathe for 6 min at a target ventilation of 85% predicted maximum voluntary ventilation (estimated as 30 × baseline FEV_1_). A dry gas mixture of 21% O_2_-5% CO_2_-balance N_2_ was delivered by a commercially available system (Eucapsys, SMTEC, Nyon, Switzerland). Ventilation was measured throughout the test, with participants receiving real-time visual feedback. The ventilation achieved during the first visit was set as the target ventilation for subsequent visits. Before and at regular time points after EVH (2, 5, 10, 15, 20, and 60 min), forced expiratory maneuvers were performed, with the maximum percent change in FEV_1_ from “baseline” (i.e., the value recorded immediately pre-EVH) used as the index for airway responsiveness. A sustained ≥10% fall in FEV_1_ (over 2 consecutive time points) was consistent with a diagnosis of EIB (4).

### Statistics

Sample size was based on previous studies that have investigated the effect of dehydration on pulmonary function (15) and EVH on airway caliber in recreationally active individuals (7, 18). Data were analyzed using statistical software (SPSS 20, SPSS, Chicago, IL). Statistical significance was set at *P* < 0.05 unless otherwise stated. Data were tested for normality using the Shapiro-Wilk test. Data for the maximum fall in FEV_1_ post-EVH were not normally distributed; therefore, differences between conditions were tested using Friedman’s two-way ANOVA by ranks test, and data are displayed as median and interquartile range (Q1–Q3). Resting spirometry, whole body plethysmography, and diffusing capacity data were normally distributed. Differences in resting pulmonary function between conditions and across times were analyzed using repeated-measures ANOVA with Bonferroni’s post hoc analysis, as needed, and data are presented as means ± SD. Heart rate and ventilation were averaged over the entire period of exercise and compared between dehydration and control conditions using paired *t*-test. Relationships between absolute changes in body mass and pulmonary function in the dehydration condition were assessed using Pearson’s correlation coefficient.

## RESULTS

### Hydration Status

Baseline body mass was not different across conditions (*P* = 0.74). The dehydration intervention caused a significant reduction in body mass (63.3 ± 10.4 kg at baseline vs. 61.8 ± 10.1 kg postexercise, *P* < 0.001), which equated to a loss of 2.3 ± 0.8%. There was no change in body mass following exercise in the control condition (63.3 ± 10.5 kg at baseline vs. 63.1 ± 10.5 kg postexercise, *P* = 0.085) or over the rest period (63.2 ± 10.8 kg at baseline vs. 63.0 ± 10.7 kg postrest, *P* = 0.12). Over the rehydration period in the dehydration condition, participants drank 830 ± 190 ml of water (61 ± 19% of the loss in body mass).

### Exercise

As expected, heart rate was significantly higher during exercise in the dehydration than control condition (148 ± 16 and 118 ± 20 beats/min, respectively, *P* < 0.001). Ventilation did not differ significantly between conditions (42 ± 15 and. 34 ± 6 l/min in dehydration and control conditions, respectively, *P* = 0.084).

### Airway Responsiveness

**Fig. 2.**
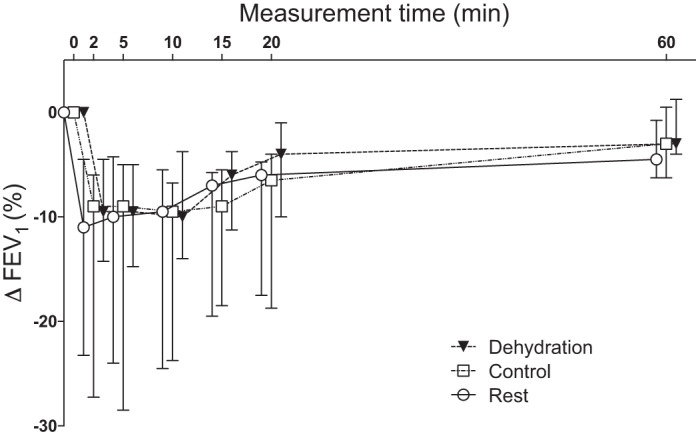
Change in forced expiratory volume in 1 s (FEV_1_) after exercise in a dehydrated state (dehydration), exercise in a euhydrated state (control), and a time-matched rest period (rest). Values are medians and interquartile ranges for 10 recreational athletes with mild asthma and/or exercise-induced bronchoconstriction.

Participants achieved a mean ventilation of 104 ± 29 l/min during the EVH challenge over the three experimental visits, which corresponded to 70 ± 9% of predicted maximum voluntary ventilation. No difference in ventilation was noted across conditions (*P* = 0.64). Seven participants (70%) had an EVH response consistent with a diagnosis of EIB in at least one condition. One additional participant had a transient fall in FEV_1_ during one visit. The median and interquartile range for maximum reduction in FEV_1_ post-EVH was 13 (7–15)%, 11 (9–24)%, and 12 (7–20)% in the dehydration, control, and rest conditions, respectively (Fig. 2). These values were not different between conditions (*P* = 0.20).

### Dynamic Lung Function

**Fig. 3.**
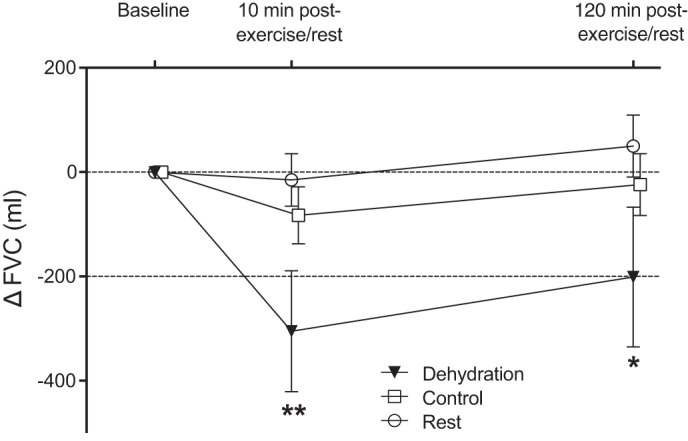
Change in forced vital capacity (FVC) after exercise in a dehydrated state (dehydration), exercise in a euhydrated state (control), and a time-matched rest period (rest). Values are means ± 95% confidence interval for 10 recreational athletes with mild asthma and/or exercise-induced bronchoconstriction. *P ≤ 0.05; **P ≤ 0.01 vs. control and rest. Reduction in FVC >200 ml (dashed lines) is considered clinically meaningful (28).

At the start of the experimental visits, pulmonary function was not different between conditions (Table 1). However, an interaction effect was noted over time between conditions (*P* < 0.001), with significant reductions in FVC only in the dehydration and control conditions (*P* < 0.001 and *P* = 0.014, respectively). In the dehydration condition, there was a sustained fall in FVC from baseline to 10 min (*P* = 0.001) and 120 min (*P* = 0.024) of recovery, while in the control condition the reduction in FVC was only transient (i.e., noted only at 10 min of recovery) (Table 1). Furthermore, the magnitude of change in FVC was greater in the dehydration condition than in the control and rest conditions (Fig. 3). In a state of dehydration, eight participants (80%) presented a clinically meaningful reduction in FVC (>200 ml), whereas only one participant in the control condition and no participants in the resting condition demonstrated a >200 ml fall in FVC. After rehydration, FVC remained slightly, but significantly, lower than baseline (−90 ± 100 ml, *P* = 0.022). No significant differences were noted between times and/or conditions for FEV_1_ and peak expiratory flow (Table 1).

**Table 1.** Dynamic lung indexes at baseline and after exercise in a dehydrated state, exercise in a euhydrated state, and a time-matched rest period

	Dehydration	Control	Rest
FEV_1_, liters			
Baseline	4.21 ± 0.89	4.17 ± 0.87	4.18 ± 0.85
10 min post	4.24 ± 0.90	4.21 ± 0.96	4.28 ± 0.90
120 min post	4.23 ± 0.89	4.24 ± 0.93	4.31 ± 0.92
Rehydrated (60 min post-EVH)	4.19 ± 0.94	4.10 ± 0.90	4.10 ± 0.81
FVC, liters			
Baseline	5.09 ± 1.22	5.09 ± 1.23	5.12 ± 1.19
10 min post	4.79 ± 1.10*^C^^,^^R^	5.00 ± 1.21*^R^	5.10 ± 1.17
120 min post	4.89 ± 1.10*^C^^,^^R^	5.06 ± 1.20	5.17 ± 1.25
Rehydrated (60 min post-EVH)	5.00 ± 1.20*	5.03 ± 1.25	5.06 ± 1.21
PEF, l/s			
Baseline	9.13 ± 2.25	9.13 ± 2.13	9.20 ± 2.10
10 min post	9.16 ± 2.01	9.47 ± 2.47	9.64 ± 2.44
120 min post	9.12 ± 2.16	9.36 ± 2.31	9.62 ± 2.32
Rehydrated (60 min post-EVH)	8.90 ± 2.17	9.10 ± 2.40	8.89 ± 1.94

Values are means ± SD for 10 participants. FEV_1_, forced expiratory volume in 1 s; FVC, forced vital capacity; PEF, peak expiratory flow; EVH, eucapnic voluntary hyperpnea.

* *P* < 0.05 vs. baseline;

C *P* < 0.05 vs. control at corresponding time point;

R *P* < 0.05 vs. rest at corresponding time point.

### Static Lung Function

**Fig. 4.**
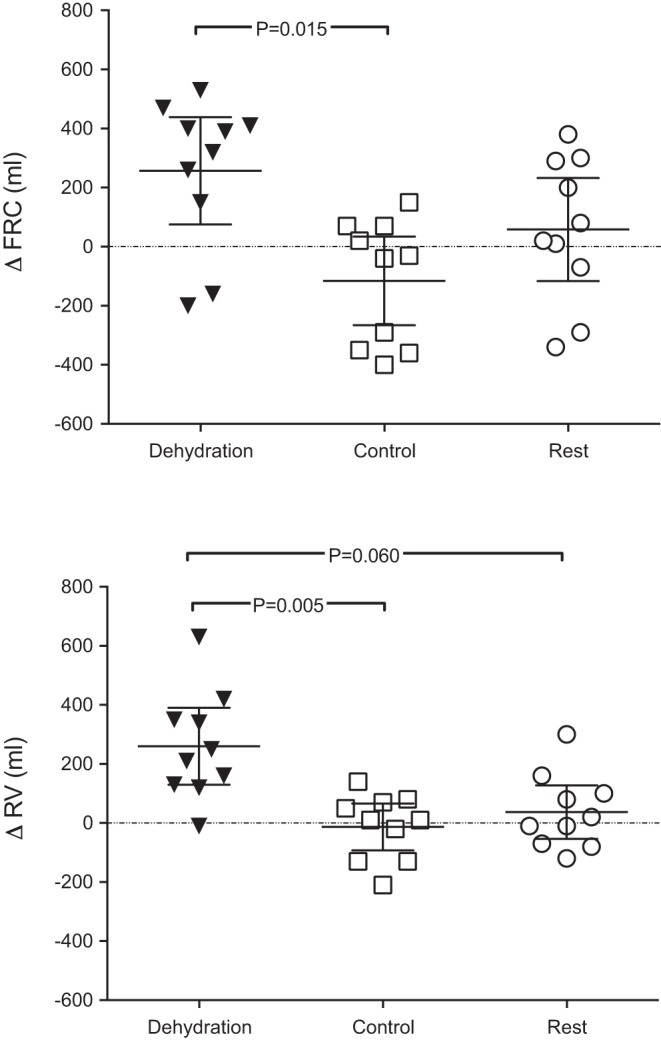
Change in functional residual capacity (FRC) and residual volume (RV) after exercise in a dehydrated state (dehydration), exercise in a euhydrated state (control), and a time-matched rest period (rest). Values are means ± 95% confidence intervals for 10 recreational athletes with mild asthma and/or exercise-induced bronchoconstriction.

Static lung volumes and capacities at baseline were not different between conditions (Table 2). Significant interaction effects were noted over the experimental conditions for FRC (*P* = 0.004) and RV (*P* = 0.001). In the dehydration condition, a significant increase in FRC was noted pre- to postexercise (260 ± 250 ml, *P* = 0.011); no difference was observed in the control or resting condition (Table 2). A concurrent increase in RV of 260 ± 182 ml was observed in the dehydration condition (*P* = 0.001; Table 2). The magnitude of change in FRC and RV from pre- to postexercise was greater in the dehydration than control condition (*P* = 0.015 and *P* = 0.060, respectively). Furthermore, the change in RV was greater in the dehydration than rest condition (*P* = 0.005; Fig. 4). No significant changes were noted between times and/or conditions for ERV or TLC (Table 2). Consequently, RV/TLC was increased under dehydration (*P* < 0.001; Table 2).

**Table 2.** Static lung volumes and capacities at baseline and after exercise in a dehydrated state, exercise in a euhydrated state, and a time-matched rest period

	Dehydration	Control	Rest
TLC, liters			
Baseline	6.70 ± 1.58	6.72 ± 1.55	6.72 ± 1.66
60 min post	6.74 ± 1.61	6.66 ± 1.62	6.71 ± 1.59
FRC, liters			
Baseline	3.40 ± 0.99	3.46 ± 1.02	3.49 ± 0.97
60 min post	3.65 ± 0.90*^C^	3.35 ± 0.95^R^	3.55 ± 1.02
RV, liters			
Baseline	1.73 ± 0.46	1.76 ± 0.45	1.77 ± 0.55
60 min post	1.99 ± 0.57*^C^	1.74 ± 0.51	1.81 ± 0.59
ERV, liters			
Baseline	1.67 ± 0.64	1.71 ± 0.67	1.72 ± 0.61
60 min post	1.67 ± 0.48	1.61 ± 0.56	1.74 ± 0.66
RV/TLC, %			
Baseline	25.9 ± 2.9	26.1 ± 2.5	26.2 ± 3.1
60 min post	29.3 ± 2.9*^C^	26.1 ± 3.0	26.8 ± 4.5

Values are means ± SD for 10 participants. TLC, total lung capacity; FRC, functional residual capacity; RV, residual volume; ERV, expiratory reserve volume.

* *P* < 0.05 vs. baseline;

C *P* < 0.05 vs. control at corresponding time point;

R *P* < 0.05 vs. rest at corresponding time point.

**Table 3.** Indexes of diffusing capacity at baseline and after exercise in a dehydrated state, exercise in a euhydrated state, and a time-matched rest period

	Dehydration	Control	Rest
Dl_CO_, mmol·min^−1^·kPa^−1^			
Baseline	10.14 ± 2.81	9.92 ± 2.69	10.16 ± 2.87
90 min post	10.07 ± 2.85	9.72 ± 2.53	9.71 ± 2.61
*K*_CO_, mmol·min^−1^·kPa^−1^·l^−1^			
Baseline	1.65 ± 0.22	1.65 ± 0.25	1.63 ± 0.27
90 min post	1.63 ± 0.20	1.60 ± 0.22	1.57 ± 0.25
Va, liters			
Baseline	6.16 ± 1.55	6.05 ± 1.45	6.13 ± 1.51
90 min post	6.18 ± 1.58	6.13 ± 1.50	6.21 ± 1.53

Values are means ± SD for 9 participants. Dl_CO_, diffusing capacity of the lung for carbon monoxide; *K*_CO_, transfer coefficient; Va, alveolar volume.

### Diffusing Capacity

There were no differences in baseline Dl_CO_, *K*_CO_, or Va between conditions. Furthermore, these variables were not modified by any of the conditions (Table 3).

### Correlation Analysis

**Fig. 5.**
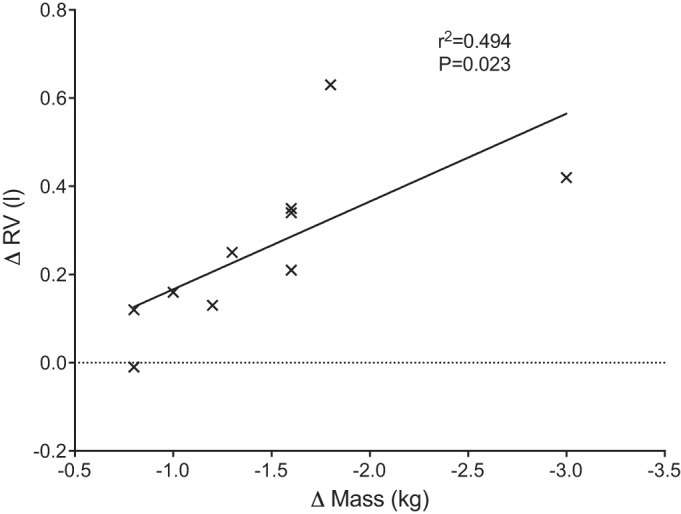
Relationship between change in body mass and change in residual volume (RV) after 2 h of exercise with fluid restriction.

There was a significant correlation (*r*^2^ = 0.494, *P* = 0.023) between the change in body mass and the change in RV at 60 min postexercise in the dehydration condition (Fig. 5). No other significant relationships were noted between study variables.

## DISCUSSION

The aim of this study was to investigate the effects of exercise-induced dehydration on airway responsiveness and pulmonary function in athletes with a medical diagnosis of mild asthma and/or EIB. We showed that mild dehydration does not increase airway responsiveness to dry air hyperpnea but is associated with alterations in lung volumes (i.e., reduced FVC and increased FRC and RV). Mild whole body dehydration is therefore unlikely to put athletes at increased risk for EIB. However, perturbations at the level of the small airways are likely to occur when athletes with preexisting lung conditions become dehydrated.

This study is the first to assess the effect of whole body dehydration on airway responsiveness. Given that athletes regularly experience exercise-induced dehydration (37) and that EIB is the most common chronic condition in elite athletes (11), these findings are highly relevant. We reasoned that whole body dehydration may have the potential to affect the volume and/or composition of airway surface liquid and, consequently, could enhance the osmotic stimulus responsible for EIB (2). However, because no difference in the severity of bronchoconstriction was noted following EVH between the dehydration and control conditions, our data do not support this hypothesis.

To maintain ecological validity, we aimed to induce a state of mild dehydration using exercise. We were successful, in that the average body mass loss was 2.3%. However, this mild degree of dehydration may have been insufficient to interfere with the pathophysiology of EIB. The volume of airway surface liquid is very small, with <0.5 ml of liquid covering the first seven generations of airways (5). Relative to the small volume of water available at the airway surface, airway water loss during hyperpnea of dry air is very high. On the basis of mathematical modeling, the net water loss within the airways during ventilation at 60 l/min in temperate conditions can exceed 0.4 ml/min (10). It is therefore possible that the large volume of respiratory water loss during EVH negated any changes in airway surface liquid induced by our dehydration protocol.

An alternative explanation for why airway responsiveness was unaffected by whole body dehydration is that EVH provoked a maximal airway response. A maximum response plateau has been shown to occur following bronchial provocation with exercise in children with asthma, with no further increase in the severity of EIB beyond 6 min of exercise (12). This raises the possibility that the use of EVH as bronchial stimulus may have masked the effects of whole body dehydration on airway responsiveness. To address this issue, future work should be conducted using a dose-response bronchial challenge, such as the mannitol test (22). Furthermore, since a maximal response plateau occurs less frequently in individuals with a greater degree of airway responsiveness (36), our findings should not be generalized to individuals with moderate-to-severe asthma/EIB.

A concurrent aim of our study was to investigate the effect of exercise-induced dehydration on basal pulmonary function. In contrast to previous research (13, 15), our results suggest that dehydration causes a reduction in FVC (with no associated change in FEV_1_). Previously, induced dehydration, by fluid deprivation (13) or diuretic drug administration (15), had no effect on FVC. However, both interventions caused a decrease (11) or an increase (13) in FEV_1_, with the latter finding attributed to a decrease in airway resistance secondary to a reduction in water content in the airway mucosa and bronchovascular sheath or a decrease in pulmonary vascular volume (13). The discrepancy in results may be due to the various protocols employed. Fluid deprivation for 16 h resulted in smaller decreases in body mass than noted in the current study [range 0.0–2.5% (13) vs. 1.5–4.4% in our study]. While a more pronounced state of dehydration was induced by diuretics (~4.5% loss of body mass) (15), the different types of water loss (hypertonic vs. isosmotic) may have influenced the results. Exercise-induced dehydration is well known to increase plasma osmolarity, whereas dehydration induced via diuretic administration generally results in isosmotic hypovolemia (33). In the present study the increase in plasma osmolarity might have caused a redistribution of fluid away from the airways, which, in turn, may have affected lung volumes. The finding of an inverse relationship between serum osmolarity and FVC in a large (>10,000) adult population (29) supports the idea that hypertonic dehydration may adversely affect pulmonary function.

In our study the reduction in FVC was associated with a concomitant increase in RV, FRC, and RV/TLC, the latter a marker of air trapping (19). Furthermore, a positive association was found between the degree of dehydration (as inferred by the reduction in body mass) and the increase in RV. Together, these results suggest that exercise-induced dehydration primarily affects the small airways. We propose that the main underlying mechanism for these changes is reduced peripheral airway stability caused by a change in the properties and/or volume of airway surface liquid in a dehydrated state. Airway surface liquid has low surface tension, which inhibits small airway closure at low lung volumes (24). If exercise-induced dehydration increases airway surface tension, it would explain the reduction in FVC and the increase in RV.

Our data show that mild exercise-induced dehydration results in sustained, clinically significant reductions in FVC [>200 ml (28)] in the majority of athletes with mild asthma/EIB. Because of controversy over a potential impairment of airway secretions in individuals with asthma (20, 26, 27), our findings may not be applicable to all athletes. Nonetheless, considering the widespread prevalence of asthma/EIB in elite athletes (11), the functional relevance of these findings deserves further attention. Because end-expiratory lung volume decreases with exercise and dehydration may affect peripheral airway stability at low lung volumes, it is tempting to speculate that exercise-induced dehydration may increase the risk of cyclic opening and closure of peripheral airways during exercise. In vitro, the reopening of closed airways can cause epithelial injury (6). As repeated epithelial injury is regarded as a key mechanism in the pathogenesis of EIB in athletes (3), these findings could be highly relevant in the context of EIB.

In conclusion, whole body dehydration does not exacerbate airway responsiveness to dry air hyperpnea in recreational athletes with mild asthma/EIB. However, lung volumes (including FVC, RV, FRC, and RV/TLC) could be compromised in a state of mild dehydration. The functional and clinical relevance of these novel findings is yet to be established.

## GRANTS

This study was supported by the European Hydration Institute Student Research Grant Scheme.

## DISCLOSURES

No conflicts of interest, financial or otherwise, are declared by the authors.

## AUTHOR CONTRIBUTIONS

A.J.S., L.M.R., and P.K. conceived and designed the research; A.J.S. performed the experiments; A.J.S. analyzed the data; A.J.S., L.M.R., and P.K. interpreted the results of the experiments; A.J.S. prepared the figures; A.J.S. drafted the manuscript; A.J.S., L.M.R., and P.K. edited and revised the manuscript; A.J.S., L.M.R., and P.K. approved the final version of the manuscript.

## References

[1] Anderson SD, Argyros GJ, Magnussen H, Holzer K Provocation by eucapnic voluntary hyperpnoea to identify exercise induced bronchoconstriction. Br J Sports Med 35: 344–347, 2001. doi:10.1136/bjsm.35.5.344. 11579071 PMC1724385

[2] Anderson SD, Daviskas E The mechanism of exercise-induced asthma is . . . . J Allergy Clin Immunol 106: 453–459, 2000. doi:10.1067/mai.2000.109822. 10984363

[3] Anderson SD, Kippelen P Exercise-induced bronchoconstriction: pathogenesis. Curr Allergy Asthma Rep 5: 116–122, 2005. doi:10.1007/s11882-005-0084-y. 15683611

[4] Anderson SD, Kippelen P Assessment of EIB: what you need to know to optimize test results. Immunol Allergy Clin North Am 33: 363–380, 2013. doi:10.1016/j.iac.2013.02.006. 23830130

[5] Anderson SD Is there a unifying hypothesis for exercise-induced asthma? J Allergy Clin Immunol 73: 660–665, 1984. doi:10.1016/0091-6749(84)90301-4. 6715730

[6] Bilek AM, Dee KC, Gaver DP 3rd Mechanisms of surface-tension-induced epithelial cell damage in a model of pulmonary airway reopening. J Appl Physiol (1985) 94: 770–783, 2003. doi:10.1152/japplphysiol.00764.2002. 12433851

[7] Bolger C, Tufvesson E, Sue-Chu M, Devereux G, Ayres JG, Bjermer L, Kippelen P Hyperpnea-induced bronchoconstriction and urinary CC16 levels in athletes. Med Sci Sports Exerc 43: 1207–1213, 2011. doi:10.1249/MSS.0b013e31820750d8. 21131866

[8] Cheuvront SN, Kenefick RW Dehydration: physiology, assessment, and performance effects. Compr Physiol 4: 257–285, 2014. doi:10.1002/cphy.c130017. 24692140

[9] Daviskas E, Gonda I, Anderson SD Mathematical modeling of heat and water transport in human respiratory tract. J Appl Physiol (1985) 69: 362–372, 1990. 2394658 10.1152/jappl.1990.69.1.362

[10] Daviskas E, Gonda I, Anderson SD Local airway heat and water vapour losses. Respir Physiol 84: 115–132, 1991. doi:10.1016/0034-5687(91)90023-C. 1852986

[11] Fitch KD An overview of asthma and airway hyper-responsiveness in Olympic athletes. Br J Sports Med 46: 413–416, 2012. doi:10.1136/bjsports-2011-090814. 22228581

[12] Godfrey S, Silverman M, Anderson SD The use of the treadmill for assessing exercise-induced asthma and the effect of varying the severity and duration of exercise. Pediatrics 56 Suppl: 893–898, 1975. 1187281

[13] Govindaraj M The effect of dehydration on the ventilatory capacity in normal subjects. Am Rev Respir Dis 105: 842–844, 1972. 5020633 10.1164/arrd.1972.105.5.842

[14] Hansen JE, Sue DY, Wasserman K Predicted values for clinical exercise testing. Am Rev Respir Dis 129: S49–S55, 1984. doi:10.1164/arrd.1984.129.2P2.S49. 6421218

[15] Javaheri S, Bosken CH, Lim SP, Dohn MN, Greene NB, Baughman RP Effects of hypohydration on lung functions in humans. Am Rev Respir Dis 135: 597–599, 1987. 3826886 10.1164/arrd.1987.135.3.597

[16] Kalhoff H Mild dehydration: a risk factor of broncho-pulmonary disorders? Eur J Clin Nutr 57 Suppl 2: S81–S87, 2003. doi:10.1038/sj.ejcn.1601906. 14681718

[17] Karjalainen J Exercise response in 404 young men with asthma: no evidence for a late asthmatic reaction. Thorax 46: 100–104, 1991. doi:10.1136/thx.46.2.100. 2014489 PMC462958

[18] Kippelen P, Larsson J, Anderson SD, Brannan JD, Delin I, Dahlén B, Dahlén SE Acute effects of beclomethasone on hyperpnea-induced bronchoconstriction. Med Sci Sports Exerc 42: 273–280, 2010. doi:10.1249/MSS.0b013e3181b541b1. 19927031

[19] Konstantinos Katsoulis K, Kostikas K, Kontakiotis T Techniques for assessing small airways function: possible applications in asthma and COPD. Respir Med 119: e2–e9, 2016. doi:10.1016/j.rmed.2013.05.003. 23764129

[20] Laitano O, Martins J, Mattiello R, Perrone C, Fischer GB, Meyer F Sweat electrolyte loss in asthmatic children during exercise in the heat. Pediatr Exerc Sci 20: 121–128, 2008. doi:10.1123/pes.20.2.121. 18579894

[21] Larsson J, Anderson SD, Dahlén SE, Dahlén B Refractoriness to exercise challenge: a review of the mechanisms old and new. Immunol Allergy Clin North Am 33: 329–345, 2013. doi:10.1016/j.iac.2013.02.004. 23830128

[22] Leuppi JD, Brannan JD, Anderson SD Bronchial provocation tests: the rationale for using inhaled mannitol as a test for airway hyperresponsiveness. Swiss Med Wkly 132: 151–158, 2002. 12070787 10.4414/smw.2002.09850

[23] Macintyre N, Crapo RO, Viegi G, Johnson DC, van der Grinten CPM, Brusasco V, Burgos F, Casaburi R, Coates A, Enright P, Gustafsson P, Hankinson J, Jensen R, McKay R, Miller MR, Navajas D, Pedersen OF, Pellegrino R, Wanger J Standardisation of the single-breath determination of carbon monoxide uptake in the lung. Eur Respir J 26: 720–735, 2005. doi:10.1183/09031936.05.00034905. 16204605

[24] Macklem PT, Proctor DF, Hogg JC The stability of peripheral airways. Respir Physiol 8: 191–203, 1970. doi:10.1016/0034-5687(70)90015-0. 5413420

[25] Miller MR, Hankinson J, Brusasco V, Burgos F, Casaburi R, Coates A, Crapo R, Enright P, van der Grinten CPM, Gustafsson P, Jensen R, Johnson DC, MacIntyre N, McKay R, Navajas D, Pedersen OF, Pellegrino R, Viegi G, Wanger J; ATS/ERS Task Force Standardisation of spirometry. Eur Respir J 26: 319–338, 2005. doi:10.1183/09031936.05.00034805. 16055882

[26] Oflu A, Soyer OU, Tuncer A, Sackesen C, Kalayci O Eccrine sweat response in children with asthma. Allergy 65: 645–648, 2010. doi:10.1111/j.1398-9995.2009.02226.x. 19886927

[27] Park C, Stafford C, Lockette W Exercise-induced asthma may be associated with diminished sweat secretion rates in humans. Chest 134: 552–558, 2008. doi:10.1378/chest.08-0366. 18641089

[28] Pellegrino R, Viegi G, Brusasco V, Crapo RO, Burgos F, Casaburi R, Coates A, van der Grinten CPM, Gustafsson P, Hankinson J, Jensen R, Johnson DC, MacIntyre N, McKay R, Miller MR, Navajas D, Pedersen OF, Wanger J Interpretative strategies for lung function tests. Eur Respir J 26: 948–968, 2005. doi:10.1183/09031936.05.00035205. 16264058

[29] Pogson ZEK, McKeever TM, Fogarty A The association between serum osmolality and lung function among adults. Eur Respir J 32: 98–104, 2008. doi:10.1183/09031936.00144207. 18321933

[30] Quanjer PH, Stanojevic S, Cole TJ, Baur X, Hall GL, Culver BH, Enright PL, Hankinson JL, Ip MSM, Zheng J, Stocks J; ERS Global Lung Function Initiative Multi-ethnic reference values for spirometry for the 3-95-yr age range: the global lung function 2012 equations. Eur Respir J 40: 1324–1343, 2012. doi:10.1183/09031936.00080312. 22743675 PMC3786581

[31] Rüst CA, Knechtle B, Knechtle P, Wirth A, Rosemann T Body mass change and ultraendurance performance: a decrease in body mass is associated with an increased running speed in male 100-km ultramarathoners. J Strength Cond Res 26: 1505–1516, 2012. doi:10.1519/JSC.0b013e318231a7b5. 22614141

[32] Sawka MN, Burke LM, Eichner ER, Maughan RJ, Montain SJ, Stachenfeld NS; American College of Sports Medicine American College of Sports Medicine position stand. Exercise and fluid replacement. Med Sci Sports Exerc 39: 377–390, 2007. 10.1249/mss.0b013e31802ca597. 17277604

[33] Sawka MN, Cheuvront SN, Kenefick RW Hypohydration and human performance: impact of environment and physiological mechanisms. Sports Med 45 Suppl 1: S51–S60, 2015. doi:10.1007/s40279-015-0395-7. 26553489 PMC4672008

[34] Spengler CM, Shea SA Endogenous circadian rhythm of pulmonary function in healthy humans. Am J Respir Crit Care Med 162: 1038–1046, 2000. doi:10.1164/ajrccm.162.3.9911107. 10988127

[35] Wanger J, Clausen JL, Coates A, Pedersen OF, Brusasco V, Burgos F, Casaburi R, Crapo R, Enright P, van der Grinten CPM, Gustafsson P, Hankinson J, Jensen R, Johnson D, Macintyre N, McKay R, Miller MR, Navajas D, Pellegrino R, Viegi G Standardisation of the measurement of lung volumes. Eur Respir J 26: 511–522, 2005. doi:10.1183/09031936.05.00035005. 16135736

[36] Woolcock AJ, Salome CM, Yan K The shape of the dose-response curve to histamine in asthmatic and normal subjects. Am Rev Respir Dis 130: 71–75, 1984. 6234831 10.1164/arrd.1984.130.1.71

[37] Zouhal H, Groussard C, Minter G, Vincent S, Cretual A, Gratas-Delamarche A, Delamarche P, Noakes TD Inverse relationship between percentage body weight change and finishing time in 643 forty-two-kilometre marathon runners. Br J Sports Med 45: 1101–1105, 2011. doi:10.1136/bjsm.2010.074641. 21160081

